# A nomogram for screening esophageal squamous cell carcinoma based on environmental risk factors in a high-incidence area of China: a population-based case-control study

**DOI:** 10.1186/s12885-021-08053-7

**Published:** 2021-03-31

**Authors:** Xiaorong Yang, Chen Suo, Tongchao Zhang, Xiaolin Yin, Jinyu Man, Ziyu Yuan, Hui Chen, Jingru Yu, Li Jin, Xingdong Chen, Ming Lu, Weimin Ye

**Affiliations:** 1 Clinical Epidemiology Unit, Qilu Hospital of Shandong University, 107 Wenhuaxi Road, Jinan, 250012 Shandong China; 2 Clinical Research Center of Shandong University, Qilu Hospital of Shandong University, Jinan, China; 3 Department of Epidemiology and Health Statistics, School of Public Health, Fudan University, Shanghai, China; 4 Department of Epidemiology and Health Statistics, School of Public Health, Shandong University, Jinan, China; 5 Fudan University Taizhou Institute of Health Sciences, Taizhou, China; 6 Department of Medical Epidemiology and Biostatistics, Karolinska Institutet, Stockholm, Sweden; 7 State Key Laboratory of Genetic Engineering, Human Phenome Institute, and School of Life Sciences, Fudan University, Songhu Road 2005, Shanghai, 200438 China; 8 Department of Epidemiology and Health Statistics & Key Laboratory of Ministry of Education for Gastrointestinal Cancer, Fujian Medical University, Fuzhou, China

**Keywords:** Esophageal squamous cell carcinoma, Environmental risk factors, Nomogram, Predictive model, Screening, Prevention

## Abstract

**Background:**

Selection of high-risk subjects for endoscopic screening of esophageal squamous cell carcinoma (ESCC) lacks individual predictive tools based on environmental risk factors.

**Methods:**

We performed a large population-based case-control study of 1418 ESCC cases and 1992 controls in a high-risk area of China. Information on potential risk factors was collected via face-to-face interview using an electronic structured questionnaire. Odds ratios (ORs) and 95% confidence intervals (CIs) were estimated using unconditional logistic regression models, and predictive nomograms were established accordingly. A weighted analysis was further conducted to introduce age into predictive nomograms due to frequency matching study design.

**Results:**

Most cases were usually exposed to 4 to 6 risk factors, but most controls were usually exposed to 3 to 5 risk factors. The AUCs of male and female predictive nomograms were 0.75 (95%CI: 0.72, 0.77) and 0.76 (95%CI: 0.73, 0.79), respectively. The weighted analysis adding age in the predictive model improved the AUC in both men and women (0.81 (95%CI: 0.79, 0.84) and 0.88 (95%CI: 0.85, 0.90), respectively).

**Conclusions:**

An easy-to-use preclinical predictive tool is provided to select candidate population with high ESCC risk for endoscopic screening. Its usefulness needs to be further evaluated in future screening practice.

**Supplementary Information:**

The online version contains supplementary material available at 10.1186/s12885-021-08053-7.

## Background

International Agency for Research on Cancer estimated that the global number of new esophageal cancer cases increased from 456,000 in 2012 to 572,034 in 2018 [1, 2], and esophageal squamous cell carcinoma (ESCC), main histopathologic subtype accounting for about 88% of esophageal cancer, remains the greatest cancer burden in some high-risk areas [3]. The incidence of ESCC varies with more than 10-fold differences across countries, and the regions with highest incidence of ESCC are concentrated in East Asia, Central Asia, the coastal zone along the Great Rift Valley, and the Gaucho Region of South America [4]. Most ESCC patients are diagnosed in late-stage and have a grim prognosis with 5-year overall survival rate of less than 25% [5, 6]. Conversely, early detection and timely treatment can improve the 5-year survival rate of early-stage ESCC to more than 80% [7, 8]. For decreasing the social burden of ESCC, the Chinese government has initiated an endoscopic screening project for esophageal cancer in several high-incidence areas [9]. Although a considerable number of early stage ESCC patients have been identified and treated with improved prognosis via the project [10, 11], less than 0.5% diagnosis rate of ESCC among all endoscopically screened populations implicates a huge waste of medical resources and low compliance in screening due to lack of a relatively accurate selection algorithm of high-risk populations [12]. The current guideline is that those having Condition 1 and any one of Conditions 2–6 should be included in high-risk group and subjected to endoscopic screening: 1) Over 40 years old; 2) From high incidence areas of esophageal cancer; 3) With upper gastrointestinal symptoms; 4) Family history of esophageal cancer; 5) With esophageal diseases; 6) With other risk factors for esophageal cancer (smoking, heavy alcohol drinking, etc.) [13]. Considering the large-scale screening project is usually conducted among asymptomatic residents, a preclinical prediction tool based on easy-to-measure environmental factors can facilitate the selection of high-risk subjects and increase the clinical compliance in field work. Hence, a quantitative prediction model which can easily output individual ESCC risk score will hopefully help risk-stratify population for targeted endoscopic screening [14].

Because more than 95% of esophageal cancer cases are ESCC in China, the current study only focuses on prediction of ESCC risk [3]. We have performed a large population-based case-control study of upper gastrointestinal cancers in Taixing, a high-incidence area in China, and have systematically assessed environmental risk factors of ESCC, including family history of esophageal cancer [15], poor hygiene [16, 17], tobacco and alcohol [18], low socioeconomic status [19], hot tea drinking [20], low BMI and high adult height [21], the interaction of genetic susceptibility and selected exposures [22, 23], and gastric atrophy [24]. However, the combined effects of candidate risk factors have not been systematically explored. Here, we aim at building an easy-to-use predictive nomogram tool of ESCC to select high-risk population based on all candidate environmental risk factors in our questionnaire, which will facilitate the selection of high-risk subjects, improve the diagnosis rate of ESCC among endoscopically screened populations, and save the limited medical resources.

## Methods

### Study design and participants

We have delineated in detail the research design of this population-based case-control study in previous reports [18, 24]. In short, we attempted to enroll all newly diagnosed esophageal cancer cases from October 2010 to September 2013 in Taixing (with a population of 1.1million), and the inclusion criteria were 40–85 year-old residents who had lived in Taixing at least 5 years. In the endoscopic units of local four largest governmental hospitals (covering almost 90% of local clinical diagnoses), potential patients were invited to complete a questionnaire by trained interviewers and provided biological samples before treatment, if they were suspected of having upper gastrointestinal tumor by endoscopic doctors. Moreover, we further enrolled missing esophageal cancer patients by cross-linkage with the local Cancer Registry. We finally recruited 1401 suspected esophageal cases from the hospitals’ endoscopy units and 280 reported esophageal cases via the local Cancer Registry during the 3 years. After reviewing the pathological sections and surgical pathology reports for those without pathological sections, 1418 ESCC patients were included in this study. We estimated that about 78.3% of the new incident cases in the research base were recruited in our study based on the statistics of the local Cancer Registry. During the same period, we randomly selected control subjects frequency-matched to ESCC cases by sex and 5-year age groups from the local Total Population Registry. Finally, 1992 eligible controls participated in our study (participation rate: 70.4%).

### Exposure assessment

All participants were interviewed face-to-face using electronic questionnaires by trained staff, which contains age, gender, race, marital status, education level, adult height at 20 years old, weight and body shape at 20 years old and 10 years ago, residence history, occupational history, family structure and family socioeconomic status, personal medical history, oral hygiene, family history of selected diseases, smoking history, passive smoking exposure, alcohol and tea drinking history, dietary history 10 years ago and female reproductive history, and so on (as shown in supplementary material).

Body mass index (BMI) (weight in kilograms divided by height in meters squared) was calculated, and subjects were categorized as underweight (< 18.5), normal weight (≥18.5 and < 24), overweight (≥24 and < 28), obesity (≥28) based on Chinese standards. The male and female adult height were converted into four categories according to the published nonlinear relationship (male cutoff values:162, 170 and 174; female cutoff values:152, 156 and 160, respectively, unit: centimeter) [21]. Family wealth score was calculated based on the ownership of valuable home items using a multiple correspondence analysis, and was further categorized as approximate quintiles among control participants [19]. The cumulative missing and filled teeth number, smoking pack-years and daily intensity of alcohol drinking among the exposed were categorized by the median.

### Statistical analysis

Analyses were stratified by gender, because of the extreme difference in prevalence and pattern of many environmental factors between men and women [20]. Chi-squared test or Kruskal–Wallis test was performed for testing the difference of the distributions of categorical variables or continuous variables between two groups. Based on all candidate environmental factors (*P* value < 0.1 in univariate analysis) in our study, we used backward elimination unconditional logistic regression model to estimate odds ratios (ORs) with 95% confidence intervals (CIs) and established a concise predictive model. The post-estimated nomogram for ESCC prediction was built to facilitate the on-site selection of high-risk population. The non-linear dose-response association of total scores with ESCC risk was assessed by restricted cubic spline regression models with five knots and the receiver operating characteristic (ROC) curve of individual score for ESCC risk was plotted to assess the accuracy of nomogram. The area under curve (AUC) was used to summarize the classification accuracy of the predictive model and 95%CI of AUC were estimated by the non-parametric bootstrap. The specificity and sensitivity were evaluated at the optimal cutoff point, which was selected using Youden’s index. Considering the significant difference of age distribution of our controls compared with local population (Table S1) due to the frequency matching case-control study design [25], we further performed a weighted analysis to introduce age as a risk factor into the regression model. All statistical analyses were conducted using Stata 15.1 (StataCorp LP, College Station, TX, USA).

## Results

The distributions of candidate environment risk factors for both healthy controls and ESCC cases stratified by gender are summarized in Table 1. Compared with control participants, male and female cases tended to have less education, lower family wealth scores, lower BMI 10 years ago, taller adult height, fewer frequency of tooth brushing per day and were more likely to have a family history of esophageal cancer among their first-degree relatives. However, since smoking, alcohol drinking and habitual tea drinking are uncommon among females, only among males, ESCC cases reported more likely to be cigarette smokers, alcohol drinkers, and hot tea drinkers than controls. Conversely, only among females ESCC cases were slightly older than controls, more likely to be farmers, and had more missing and filled teeth. Among controls, males differed significantly from females regarding several characteristics, i.e., marital status, occupation, education level, BMI, adult height, sum of missing and filled teeth, smoking, alcohol drinking and tea drinking. After summarizing the number of candidate environment risk factors, most cases were exposed to 4 to 6 risk factors, while most controls were exposed to 3 to 5 risk factors.

**Table 1:** Demographic Information of the Study Subjects Enrolled in a Population-based Case-control Study of Esophageal Squamous Cell Carcinoma, Taixing, China, 2010–2013, stratified by gender

Variables	Men			Women			***P*** value for comparing male and female controls ^**b**^
	Controls (***N***=1373) N (%)	Cases (***N***=962) N (%)	***P*** value ^a^	Controls (***N***=619) N (%)	Cases (***N***=456) N (%)	***P*** value ^a^	
**Age at interview (mean±SD, years)**	65.6**±**8.4	65.3**±**8.5	0.228	67.6**±**9.4	69.4**±**7.6	0.024	0.0001
**Age group**							
40-49	53 (3.86)	30 (3.12)	0.134	29 (4.68)	5 (1.1)	<0.001	<0.001
50-59	277 (20.17)	215 (22.35)		90 (14.54)	35 (7.68)		
60-69	586 (42.68)	417 (43.35)		209 (33.76)	187 (41.01)		
70-79	407 (29.64)	252 (26.2)		237 (38.29)	187 (41.01)		
80-84	50 (3.64)	48 (4.99)		54 (8.72)	42 (9.21)		
**Marital status**							
Unmarried	63 (4.59)	55 (5.72)	0.278	5 (0.81)	2 (0.44)	0.575	<0.001
Married	1160 (84.49)	790 (82.12)		428 (69.14)	307 (67.32)		
Divorced/Widowed	150 (10.92)	117 (12.16)		186 (30.05)	147 (32.24)		
**Occupation**							
Farmer	713 (51.93)	517 (53.74)	0.673	547 (88.37)	431 (94.52)	0.002	<0.001
Worker	356 (25.93)	243 (25.26)		45 (7.27)	17 (3.73)		
Service/Clerk/Professional/Administrator	304 (22.14)	202 (21)		27 (4.36)	8 (1.75)		
**Education**							
Illiteracy	171 (12.45)	169 (17.57)	0.002	369 (59.61)	329 (72.15)	<0.001	<0.001
Primary school	589 (42.9)	419 (43.56)		171 (27.63)	109 (23.9)		
Junior high school	463 (33.72)	285 (29.63)		67 (10.82)	16 (3.51)		
High school and above	150 (10.92)	89 (9.25)		12 (1.94)	2 (0.44)		
**Family wealth score**							
Q1-lowest	288 (20.98)	282 (29.31)	<0.001	120 (19.39)	122 (26.75)	0.024	0.351
Q2	234 (17.04)	166 (17.26)		119 (19.22)	89 (19.52)		
Q3	289 (21.05)	216 (22.45)		145 (23.42)	106 (23.25)		
Q4	297 (21.63)	178 (18.5)		132 (21.32)	84 (18.42)		
Q5	265 (19.3)	120 (12.47)		103 (16.64)	55 (12.06)		
**BMI at 10 years ago**							
<18.5 (Underweight)	57 (4.15)	71 (7.38)	<0.001	53 (8.56)	61 (13.38)	0.005	<0.001
18.5-24) (Normal)	864 (62.93)	652 (67.78)		341 (55.09)	251 (55.04)		
[24, 28) (Overweight)	367 (26.73)	195 (20.27)		178 (28.76)	127 (27.85)		
≥ 28 (Obese)	83 (6.05)	41 (4.26)		46 (7.43)	16 (3.51)		
Missing	2 (0.15)	3 (0.31)		1 (0.16)	1 (0.22)		
**Adult height (cm, male | female)**							
≤ 162 | ≤ 152	500 (36.42)	164 (17.05)	<0.001	244 (39.42)	66 (14.47)	<0.001	<0.001
(162, 170] | (152, 156]	637 (46.39)	513 (53.33)		153 (24.72)	154 (33.77)		
(170-174] | (156, 160]	118 (8.59)	131 (13.62)		141 (22.78)	152 (33.33)		
>174 | >160	116 (8.45)	151 (15.7)		80 (12.92)	83 (18.2)		
Missing	2 (0.15)	3 (0.31)		1 (0.16)	1 (0.22)		
**Frequency of tooth brushing per day**							
< 2	903 (65.77)	758 (78.79)	<0.001	387 (62.52)	352 (77.19)	<0.001	0.141
≥ 2	452 (32.92)	174 (18.09)		225 (36.35)	87 (19.08)		
Missing	18 (1.31)	30 (3.12)		7 (1.13)	17 (3.73)		
**Sum of missing and filled teeth**							
None	370 (26.95)	245 (25.47)	0.177	130 (21.00)	59 (12.94)	0.001	<0.001
< 6	514 (37.44)	327 (33.99)		203 (32.79)	132 (28.95)		
≥ 6	471 (34.3)	358 (37.21)		278 (44.91)	240 (52.63)		
Missing	18 (1.31)	32 (3.33)		8 (1.29)	25 (5.48)		
**Smoking pack-years**							
Never	299 (21.78)	153 (15.9)	<0.001	585 (94.51)	413 (90.57)	0.875	<0.001
≤ 30	522 (38.02)	327 (33.99)		20 (3.23)	15 (3.29)		
> 30	531 (38.67)	438 (45.53)		4 (0.65)	4 (0.88)		
Missing	21 (1.53)	44 (4.57)		10 (1.62)	24 (5.26)		
**Alcohol drinking intensity (g/day)**							
Never	584 (42.53)	235 (24.43)	<0.001	574 (92.73)	400 (87.72)	0.306	<0.001
≤ 80	373 (27.17)	289 (30.04)		31 (5.01)	31 (6.8)		
> 80	395 (28.77)	397 (41.27)		3 (0.48)	1 (0.22)		
Missing	21 (1.53)	41 (4.26)		11 (1.78)	24 (5.26)		
**Tea drinking temperature**							
Never	858 (62.49)	493 (51.25)	<0.001	578 (93.38)	420 (92.11)	0.199	<0.001
Warm	206 (15)	150 (15.59)		19 (3.07)	11 (2.41)		
Hot	213 (15.51)	176 (18.3)		8 (1.29)	1 (0.22)		
Very Hot	76 (5.54)	102 (10.6)		4 (0.65)	2 (0.44)		
Missing	20 (1.46)	41 (4.26)		10 (1.62)	22 (4.82)		
**Family history of esophageal cancer among first-degree relatives**							
No	1106 (80.55)	632 (65.7)	<0.001	499 (80.61)	296 (64.91)	<0.001	0.905
Yes	252 (18.35)	298 (30.98)		112 (18.09)	141 (30.92)		
Missing	15 (1.09)	32 (3.33)		8 (1.29)	19 (4.17)		
**Number of risk factors** ^**c**^							
0 risk factor	8 (0.58)	0 (0)	<0.001	1 (0.16)	0 (0)	<0.001	<0.001
1 risk factor	48 (3.5)	13 (1.35)		7 (1.13)	1 (0.22)		
2 risk factors	167 (12.16)	34 (3.53)		32 (5.17)	5 (1.1)		
3 risk factors	335 (24.4)	116 (12.06)		81 (13.09)	16 (3.51)		
4 risk factors	402 (29.28)	223 (23.18)		184 (29.73)	72 (15.79)		
5 risk factors	263 (19.16)	267 (27.75)		197 (31.83)	153 (33.55)		
6 risk factors	96 (6.99)	198 (20.58)		94 (15.19)	133 (29.17)		
7 risk factors	22 (1.6)	50 (5.2)		10 (1.62)	47 (10.31)		
8 risk factors	2 (0.15)	11 (1.14)		4 (0.65)	2 (0.44)		
Missing	30 (2.18)	50 (5.2)		9 (1.45)	27 (5.92)		

Abbreviations: *SD* standard deviation, *N* number, *BMI* body mass index

^a^ *P* values were derived using Kruskal–Wallis test for continuous variables and Chi-squared test or Fisher exact test for categorical variables, after excluding the corresponding missing values

^b^ *P* values were for comparisons between male and female controls

^c^ risk factor were defined as low education level (illiteracy, primary school), family wealth score (Q1, Q2, Q3), BMI (underweight), tooth brushing times (< 2), smoking pack-years (> 0), alcohol consumption intensity (> 0), tea drinking temperature (hot, very hot), and family history of esophageal cancer among first-degree relatives (yes), occupation (farmer), sum of missing and filled teeth (> 0), which were defined based on results from univariate analysis (*P* < 0.05)

**Fig. 1:**
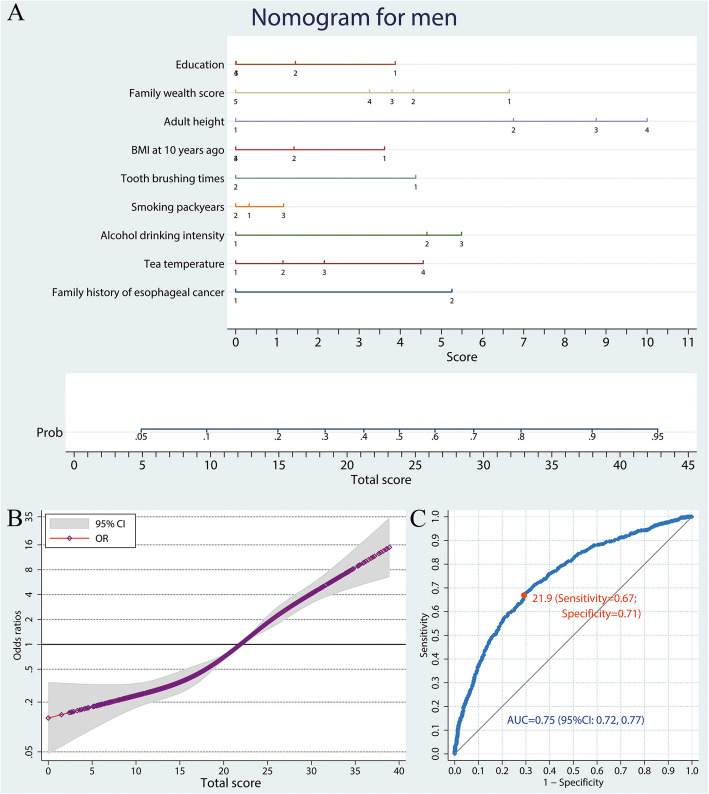
A nomogram to predict ESCC risk in men. **a** Predictive nomogram of ESCC. Points corresponding to each category of variables are listed in Table 1. **b** Non-linear dose-response relation between total scores and ESCC risk. The model was adjusted for age and the reference was set as total score of 21.9. **c** ROC curve of the nomogram model. ESCC, esophageal squamous cell carcinoma; OR, odds ratio; ROC, receiver-operating characteristic; AUC, area under curve

The candidate variables identified by univariate analysis were all included in the predictive model for males. Namely, for males, the predictive nomogram distinguishing ESCC cases from healthy controls included education, family wealth score, BMI, adult height, tooth brushing times, smoking pack-years, alcohol drinking intensity, tea drinking temperature, and family history of esophageal cancer (Fig. 1a). For each participant, points were assigned for each category of independent ESCC risk factors, then a total score and a corresponding predicted probability of developing ESCC were calculated from the nomogram. The non-linear dose-response association of total scores with ESCC risk is illustrated in Fig. 1b, with a significant monotonous increasing trend. A ROC curve was plotted to estimate the predictive accuracy of the nomogram, and the corresponding AUC (95% CI) was 0.75 (0.72–0.77; Fig. 1c). The ORs (95% CI) and points for all predictive variables are listed in Table S2.

**Fig. 2:**
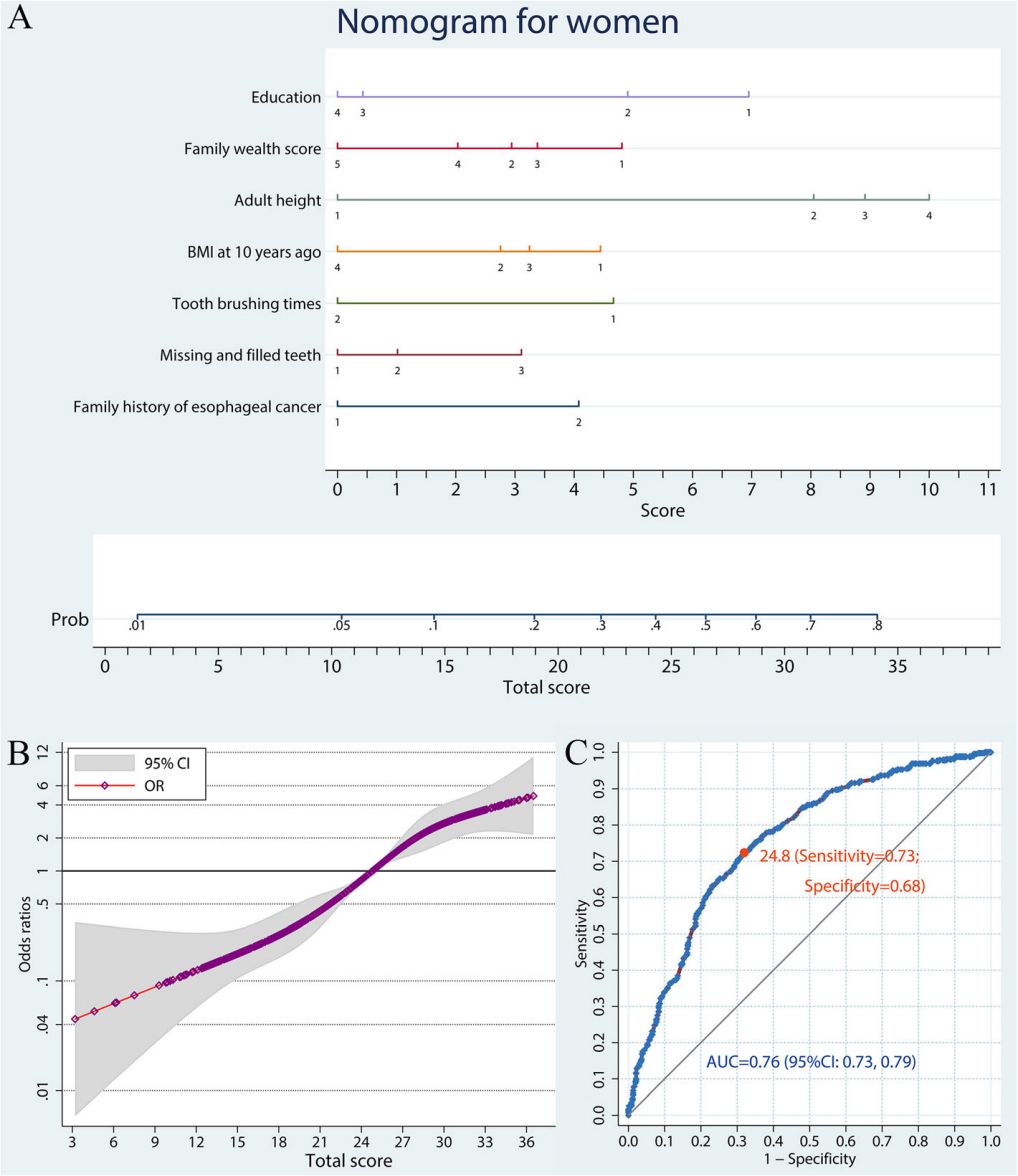
A nomogram to predict ESCC risk in women. **a** Predictive nomogram of ESCC. Points corresponding to each category of variables are listed in Table 1. **b** Non-linear dose-response relation between total scores and ESCC risk. The model was adjusted for age and the reference was set as total score of 24.8. **c** ROC curve of the nomogram model. ESCC, esophageal squamous cell carcinoma; OR, odds ratio; ROC, receiver-operating characteristic; AUC, area under curve

The variable occupation was removed from the predictive model for females, because of its collinearity with education and family wealth score. Thus the nomogram to predict ESCC risk among females included education, family wealth score, BMI, adult height, tooth brushing times, missing and filled teeth number, and family history of esophageal cancer (Fig. 2a, Table S2). The monotonous increase of ESCC risk in association with total scores in women is illustrated in Fig. 2b. The AUC (95% CI) of the nomogram predictive tool for females was 0.76 (0.73–0.79; Fig. 2c).

### Weighted analysis

**Fig. 3:**
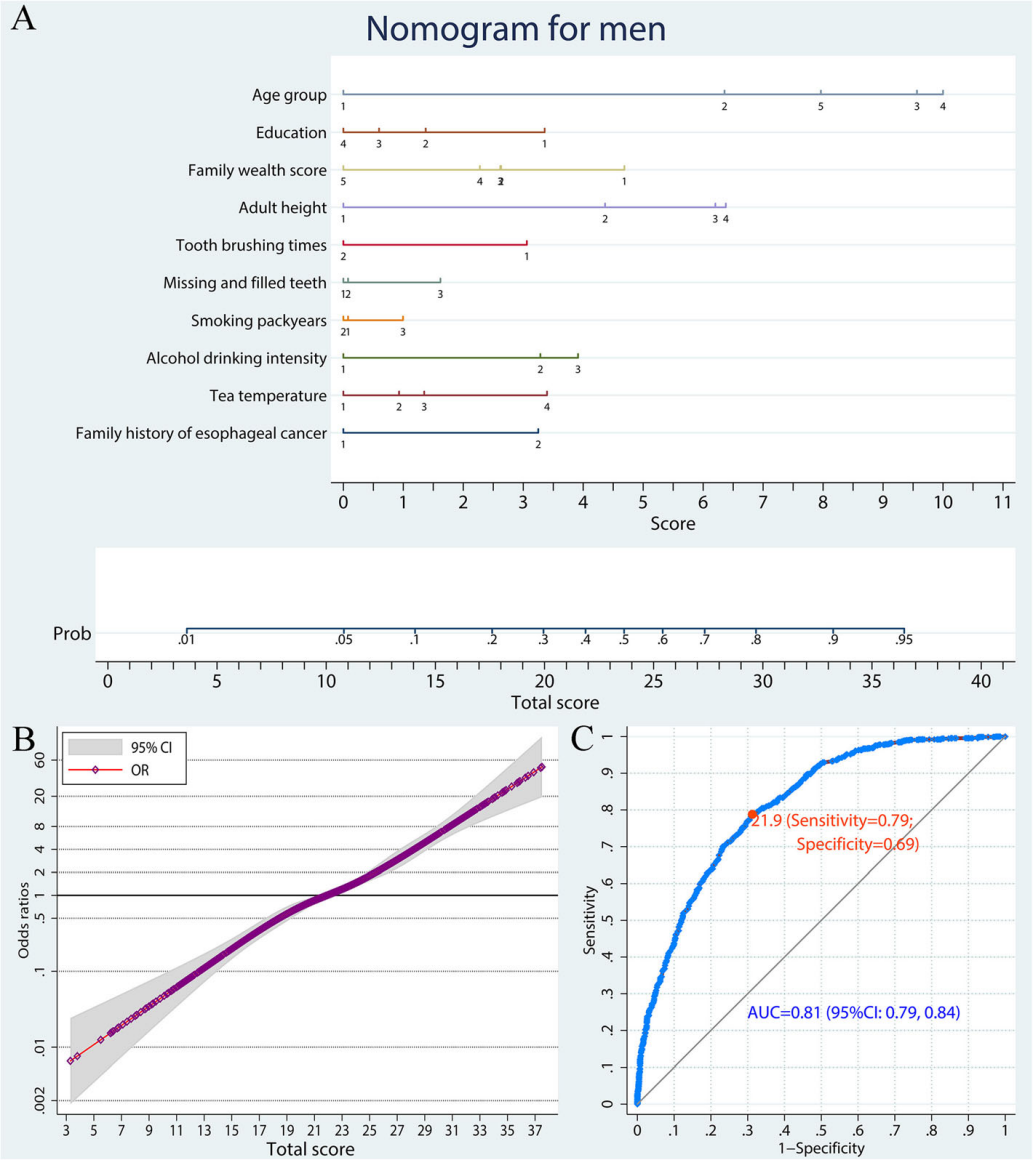
A nomogram to predict ESCC risk in men by a weighted analysis. **a** Predictive nomogram of ESCC. Points corresponding to each category of variables are listed in Table 1. **b** Non-linear dose-response relation between total scores and ESCC risk. The reference was set as total score of 21.9. **c** ROC curve of the nomogram model. ESCC, esophageal squamous cell carcinoma; OR, odds ratio; ROC, receiver-operating characteristic; AUC, area under curve

Through the weighted analysis adjusting the age disparity between controls and the general population, risk factors included in the prediction model for males contained age group, education, family wealth score, adult height, frequency of tooth brushing, missing and filled teeth, smoking pack-years, alcohol drinking intensity, tea drinking temperature, and family history of esophageal cancer (Fig. 3a, Table S3). The monotone increasing risk of ESCC with increasing total scores is illustrated in Fig. 3b. The AUC of ROC curve for the prediction model for males was 0.81 (95% CI: 0.79, 0.84; Fig. 3c).

**Fig. 4:**
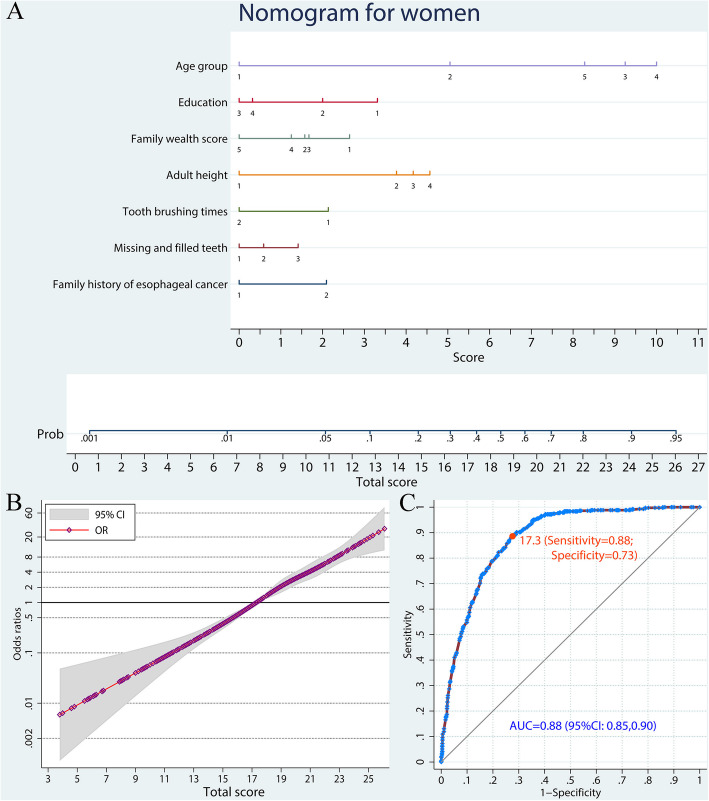
A nomogram to predict ESCC risk in women by a weighted analysis. **a** Predictive nomogram of ESCC. Points corresponding to each category of variables are listed in Table 1. **b** Non-linear dose-response relation between total scores and ESCC risk. The reference was set as total score of 17.3. **c** ROC curve of the nomogram model. ESCC, esophageal squamous cell carcinoma; OR, odds ratio; ROC, receiver-operating characteristic; AUC, area under curve

Analogously, age, education, family wealth score, adult height, tooth brushing frequency, missing and filled teeth number, and family history of esophageal cancer were included in the predictive model for females, with an AUC of 0.88 (95% CI: 0.85–0.90; Fig. 4, Table S3).

## Discussion

Several prospective studies have demonstrated that endoscopic screening for early detection of ESCC could reduce its mortality [26–28]. However, among those endoscopically screened, the detection rate of ESCC cases is less than 0.5%, resulting in low cost-efficiency of ESCC screening programs due to lack of accurate risk prediction tools for risk-stratification [11, 12]. Moreover, field experiences show that endoscopic screening with a low true positive rate leads to poor compliance in the preselected population. A quantitative predictive tool providing individual risk assessment could help candidates make reasonable decision on whether or not to undergo endoscopy examination.

Recently, Sheikh et al. generated a risk scoring system summing up exposures like smoking, hot tea drinking, fruit intake, vegetable intake, tooth loss, un-piped water, and indoor air pollution and presented a significant dose-dependent relationship between ESCC risk and combined environmental risk factors based on the Golestan Cohort Study in Iran [29], but it was not suitable for preselection of high-risk population, because the individual ESCC risk probability was not evaluated. A nomogram for predicting the risk of mixed premalignant lesions containing reflux esophagitis, inflammatory lesions, dysplasia, and so on, showed an AUC of 0.749 (0.711–0.788) based on information on age, sex, education, occupation, income, labor intensity and mining exposure collected from an esophageal endoscopic screening project in China [12], but the etiology of ESCC is substantially different from esophagitis and mixed premalignant lesions [30, 31]. As a risk prediction method, nomogram has shown promising value in clinical prognosis prediction [32, 33]. Here, to our best knowledge, we first established an easy-to-use prediction tool via nomogram to optimize the preselection of candidate high-risk population for ESCC endoscopic screening programs.

The relationships between all identified environmental risk factors and ESCC risk have been well discussed in our previous articles [15, 17–21]. Although several predictive variables may not directly cause ESCC, they might be surrogate variables and their predictive values are notable [34, 35]. For primary prevention of ESCC, promoting good oral hygiene and alcohol abstinence should be the most cost-efficient and easy-to-apply community intervention measures. Moreover, based on our results, we will develop a mobile App to automatically analyze and report the individual ESCC risk when the information of multiple environmental risk factors is collected. If asymptomatic residents receive a high score of ESCC development probability, they are advised to avoid risk factors, and also can choose to undergo a prophylactic endoscopic examination.

Our study has several advantages. To decrease selection bias, we attempted to enroll all newly-diagnosed ESCC cases and randomly selected frequency matching control participants from the total registry of residents of the study area. We interviewed most ESCC cases before they were aware of their diagnoses, which would partly reduce potential report bias and recall bias. Moreover, our study had a relatively large sample size, independent pathophysiological confirmation of all cases, relatively high response rates for both cases and controls, and the systematic collection of information on environmental risk factors.

There are also some limitations to our nomogram-based model. First, the study was conducted in a high-risk area of ESCC in China, which would weaken the generalization of our risk prediction tool to other areas. Second, despite our best efforts to collect candidate risk factors of ESCC, the questionnaire interview hardly covered all information of ESCC etiological factors. Third, although the AUCs of ESCC prediction nomogram in both sexes were slightly over 0.8, the predictive tool was not generated to deliver an accurate diagnosis but to optimize the preselection of eligible high-risk population of ESCC for endoscopic screening which is better than available approaches at present. Finally, the age distribution of the frequency matching controls was different from that of local residents, but we performed a weighted analysis to overcome this limitation.

## Conclusions

We established an easy-to-use preclinical prediction tool for both sexes to select candidate population with high ESCC risk for subsequent endoscopy screening, which optimizes the implementation of endoscopic screening projects and promotes early prevention of ESCC. The diagnostic accuracy and cost-effectiveness of our predictive tool need to be further validated in prospective studies and reappraised in ESCC screening programs.

## Supplementary Information

**Additional file 1: Supplementary material.** The questionnaire of upper gastrointestinal disease in Taixing, China.

**Additional file 2: Table S1.** The age distribution of local residents in Taixing city and the weight of controls in our data analysis.

**Additional file 3: Table S2.** OR with 95%CIs and nomogram points of candidate ESCC risk factors.

**Additional file 4: Table S3.** OR with 95%CIs and nomogram points of candidate ESCC risk factors by a weighted analysis.

## Data Availability

All data that support the findings of this study are available from the corresponding authors for a reasonable request.

## Abbreviations

ESCC: Esophageal squamous cell carcinoma
BMI: Body mass index
OR: Odds ratios
CI: Confidence intervals
ROC: Receiver operating characteristic
AUC: Area under curve
