# Supplementary material for: A nomogram for screening esophageal squamous cell carcinoma based on environmental risk factors in a high-incidence area of China: a population-based case-control study
Source: BMC Cancer. 2021 Mar 31;21:343. doi: 10.1186/s12885-021-08053-7 (PMC8011400; doi:10.1186/s12885-021-08053-7)
Supplement: Supplementary file 1 — Additional file 1: Supplementary material. The questionnaire of upper gastrointestinal disease in Taixing, China. [file 12885_2021_8053_MOESM1_ESM.pdf]

Labeling

Main Questionnaire of upper gastrointestinal disease

2010

|__|__|__|__|—|__||__|—|__|—|__|__|__|__|—|__|—|__|

Region Year Group Serial number Sample Branch

Date of diagnosis: ___Year___Month __Day (cases)

Diagnostic hospital: _________________ (cases)

Inpatient (outpatient) number:_________ (cases)

Pathology No: _____(cases) Name of diagnosis____________

Date of visit:_____ Year____ Month____ day

Start time:__ Hour__ Minute

End time: __ Hour__ Minute

End question:_____

Code Description:

1. **Region: TXRY (Taixing Renmin Hospital)/ TXEY (Taixing Second Hospital) / TXHQ (Taixing Huangqiao Hospital / TXSY (Taixing Third Hospital);**
2. **Year: the year when the subject was investigated;**
3. **Group: A-esophageal cancer B-gastric cancer C-liver cancer D-controls**
4. **Serial number: the independent serial number of the subject, which is filled in by the investigator according to the specimen label;**
5. **Sample: the questionnaire is I;**
6. **Branch: the branch number is 1.**

Before I start, I'd like to make three points for you

1. You may not remember all the questions we asked you, but all of them are very useful to us. I will ask you questions in the order arranged in advance. If you encounter some questions that have already been answered, please give me a complete answer. If you really don't know some questions, please answer "no".
2. If you have other questions to ask, please wait for me to ask all the questions, and I will answer them for you.
3. The content of this questionnaire and your name will not be released.

Part I. Basic Information

1.1. Name: ________________________________

1.2 Identification number: |__|__|__|__|__|__|__|__|__|__|__|__|__|__|__|__|__|__|

1.3. Tel: |__|__|__|__|(state code) - |__|__|__|__|__|__|__|__|

|__|__|__|__|__|__|__|__|__|__|__| (cell phone number)

1.4. Residence Address: __________ Province _________City/County__________________________

1.5. Current Address: __________ Province _________City/County____________________________

Postal Code: |__|__|__|__|__|__|

1.5.1 Please provide the name of your relative so that you can contact the relative when you cannot be contacted

Name of relativ: ____________________________ Relationship: _______________

Tel: |__|__|__|__|(state code) - |__|__|__|__|__|__|__|__|

|__|__|__|__|__|__|__|__|__|__|__| (cell phone number)

Current Address: __________ Province _________City/County__________________________

1.6. Sex: |__| (Male =1, Female =2)

1.7.Birthday : |__|__|__|__| / |__|__| / |__|__| (based on identification number)
Year Month Day

1.8. Age |__|__| years old

1.9. Nationality: |__| (1)Han (2)Hui (3)Other___________

1.11 Native place: ____________ Province _________City/County

1.12 Native place of mother: ___________ Province _________City/County

1.13 Marital Status : |__| 1 - unmarried 2 - married 3 - divorced 4 - widowed

1.14 Education: |__| 1- illiteracy 2- primary school 3- junior high school 4- senior high school / technical secondary school 5- junior college 6- bachelor degree or above

1.15 Height at age 20 years old ____ (centimeter)

1.16 Weight at about age 20 years old ____(kg)

1.17 Weight 10 years ago____(kg)

1.18 Body shape of different life periods (see the following figure):

<u>Male</u>

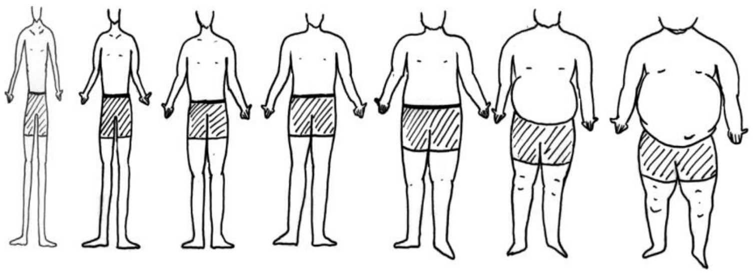

1 2 3 4 5 6 7

1.18.1 About 20 years old: |__|

1.18.2 10 years ago: |__|

1.18.3 Present: |__|

<u>Female</u>

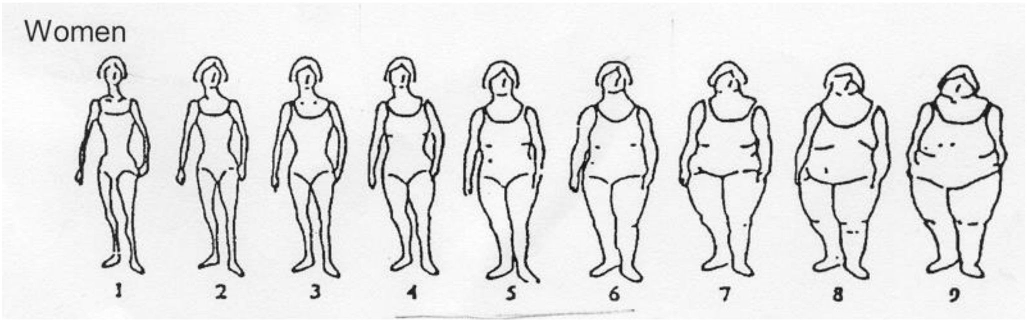

1.19.1 About 20 years old: |__|

1.19.2 10 years ago: |__|

1.19.3 Present: |__|

Part II. Residential history

Please list all residences, cooking appliances and fuel types that have lived for more than one year, starting from birth.

N	From (yeras old)	To (yeras old)	Residence (1) urban (2) rural	City	Village	House type	Cooking fuel
1	|__||__|	|__||__|	____________|__|			|__|	|__|
2	|__||__|	|__||__|	____________|__|			|__|	|__|
3	|__||__|	|__||__|	____________|__|			|__|	|__|
4	|__||__|	|__||__|	____________|__|			|__|	|__|
5	|__||__|	|__||__|	____________|__|			|__|	|__|
6	|__||__|	|__||__|	____________|__|			|__|	|__|
7	|__||__|	|__||__|	____________|__|			|__|	|__|
8	|__||__|	|__||__|	____________|__|			|__|	|__|

Notes:
House type: 1 = wooden brick structure, 2 = adobe house, 3 = brick concrete structure, 4 = building, 5 = others (please specify) _____ 6 = don't know

Main heating devices: 1 = no, 2 = stove with chimney, 3 = stove without chimney, 4 = air conditioner, 5 = others (please specify)_____ 6 = don't know

Cooking fuel: 1 = gas, 2 = carbon, coal, 3 = electricity, 4 = wood, 5 = traditional stove, 6 = others (please specify)_____ 7 = don't know

Part III. Occupational history

The following is about the work situation and what chemical substances have been exposed to in the working environment

3.1 How old were you when you started working full-time [more than 20 hours a week]? |__|__| (If you have no job: 98)

3.2 How old were you when you stopped working full-time? |__|__| (If you are still working, write down the present age)

3.3 please list all the jobs you have been engaged in for more than one year (from recent to early work, including housework), and the labor intensity in the work:

N	From (yeras old)	To (yeras old)	Job name	Labor intensity
1	|__|__|	|__|__|	|__|__|	|__|__|
2	|__|__|	|__|__|	|__|__|	|__|__|
3	|__|__|	|__|__|	|__|__|	|__|__|
4	|__|__|	|__|__|	|__|__|	|__|__|
5	|__|__|	|__|__|	|__|__|	|__|__|
6	|__|__|	|__|__|	|__|__|	|__|__|
7	|__|__|	|__|__|	|__|__|	|__|__|
8	|__|__|	|__|__|	|__|__|	|__|__|

Job name: Occupation Classification Code

Code	Content	Code	Content	Code	Content	Code	Content
**0**	Unknown	**17**	Job hunting	**34**	Pastoral workers	**51**	Paper products
**01**	Scientific researchers	**18**	Unemployed	**35**	Fishery workers	**52**	Silver products
**02**	Industrial and agricultural technicians	**19**	Administrative staff	**36**	Hunting workers	**53**	Stone cutter and Carver
**03**	Technology management and support	**20**	Political and security personnel	**37**	Agricultural machinery operators	**54**	Installation and operation of forging machine
**04**	Aircraft and marine technician	**21**	Posts and Telecommunications staff	**38**	Other agricultural, animal husbandry and fishery personnel	**55**	Machine assembly and instrument manufacturing
**05**	Health professionals	**22**	Other clerks	**39**	Factory foreman	**56**	Electronic installation, repair and assembly
**06**	Business personnel	**23**	Salesmen	**40**	Drilling technology for mining halite exploration	**57**	Radio recording projectionist

**07**	Legal staff	**24**	Purchasing, supply and marketing personnel	**41**	Metal smelting processing	**58**	Pipe welding
**08**	Teaching staff	**25**	Acquisition personnel	**42**	chemical products	**59**	Glass ceramic products
**09**	Arts and sports personnel	**26**	Other business personnel	**43**	Rubber and plastic products	**60**	Painter
**10**	Cultural staff	**27**	Waiter	**44**	Textile printing and dyeing operations	**61**	Other production workers
**11**	Religious professionals	**28**	Chefs and cooks	**45**	Leather and fur products	**62**	Construction worker
**12**	Heads of state agencies	**29**	Tour guide	**46**	Tailoring and sewing	**63**	Power equipment operator
**13**	Head of Party and Mass Organization	**30**	Daily necessities repaire	**47**	Food and beverage products	**64**	Loading and unloading operators
**14**	Person in charge of enterprises and institutions	**31**	Other service personnel	**48**	Tobacco workers	**65**	Transport equipment analyst
**15**	Students	**32**	Agricultural workers	**49**	Incense workers	**66**	Inspection metrological analyst
**16**	Housework	**33**	Forestry workers	**50**	Rattan palm grass worke	**67**	Other production and transportation personnel

Labor intensity: 1= jobs that can be done while sitting; 2= jobs that require minimal effort such as those done standing, sitting or with slow walk, that do not require much physical effort (e.g., shop assistant, hairdresser, guard, etc.); 3= jobs that require carrying light loads, continuous working, mainly indoor activities and that would occasionally increase the heart rate slightly and cause light perspiration (e.g., cleaner, nurse, electrician, etc.); 4= jobs that require carrying heavy loads, brisk walking, mainly outdoor activity, that increase heart rate substantially and cause heavy sweating (e.g., miner, bricklayer, construction worker, shoveling, etc.)

For women (Enter 98 for men):

3.4 If married, what is the current occupation of your husband? (If unmarried, what is the current occupation of the head of household?) |__||__|

Part IV. Information about family and SES

4.1 4.1 The place where you are living in: |__|

(1) belongs to you (2) you are renting

(3) has been provided by the government, or a company where you work

(4) belongs to your parents (5) Other (specify) ____________________.

4.2 What is the area of your place of residence (in square meters) excluding garden, terrace, rooms for animals, workshops, etc.? |__||__||__||__|

4.3 How many family members live together in your current house? |__||__|

4.4 what is the highest level of education of the head of your household? |__|

(1) Nil (2) Less or equal than 5th Std. (3) 6th-8th Std.

(4) 9-12 Std. (5) College / Graduation

4.5. Which of the following facilities is available in your household?

	**Facilities**	**(1)Yes (2) No**	**If Yes, for how long have you owned one (in years) (No =98)**
4.5.1	Personal automobile	|__|	|__||__|
4.5.2	Personal motorcycle	|__|	|__||__|
4.5.3	Color TV	|__|	|__||__|
4.5.4	Black and white TV	|__|	|__||__|
4.5.5	Bath inside your house	|__|	|__||__|
4.5.6	Vacuum cleaner	|__|	|__||__|
4.5.7	Washing machine	|__|	|__||__|
4.5.8	Refrigerator	|__|	|__||__|
4.5.9	Freezer	|__|	|__||__|
4.5.10	Computer	|__|	|__||__|

Part V. Personal history of disease

* Notes: Diseases should be diagnosed at least by a town hospital. Being cured was defined as not longer taking the medications.

5.1 personal disease history:

	Disease	(1)Yes (2)No	Age at first diagnosis (No = 98)
5.1.1	Rheumatic heart disease	|__|	|__||__|
5.1.2	Angina/ infarction / heart failure	|__|	|__||__|
5.1.3	Stroke	|__|	|__||__|
5.1.4	Hypertension	|__|	|__||__|
5.1.5	Diabetes	|__|	|__||__|
5.1.6	COPD (Asthma, chronic bronchitis, emphysema)	|__|	|__||__|
5.1.7	Chronic renal failure	|__|	|__||__|
5.1.8	Jaundice	|__|	|__||__|
5.1.9	Chronic liver disease	|__|	|__||__|
5.1.10	Tuberculosis	|__|	|__||__|

5.2 Drug history

5.2.1 have you used a certain drug (Western Medicine) regularly? |__| (1) Yes (2) No

If yes, please list the drug name and frequency of use (all drugs including contraceptives and vitamins)

Drug name	Present status (1) Yes (2) No	Use time (year)	Frequency of use *	dose
Contraceptives	|__|	|__||__|	|__|	
**Vitamins**	|__|	|__||__|	|__|	
Aspirin	|__|	|__||__|	|__|	
Others ___________	|__|	|__||__|	|__|	
Others ___________	|__|	|__||__|	|__|	
Others ___________	|__|	|__||__|	|__|	

***** Frequency of use: (1) every day or more than 20 times a month (2) weekly (3) monthly or less (for long-term treatment, such as 3-4 weeks)

5.2.2 Do you often take Chinese herbal medicine? |__| (1) Yes (2) No

**Indications**	**Have you ever used it**<br>**(1) Yes (2) No**	**Use time (year)**	**Frequency of use ***
Cold clearing and fever relieving	|__|	|__|__|	|__|
Dispelling wind	|__|	|__|__|	|__|
Clearing away heat and toxin	|__|	|__|__|	|__|
Dispelling phlegm and relieving cough	|__|	|__|__|	|__|
Promoting blood circulation and removing blood stasis	|__|	|__|__|	|__|
Conditioning	|__|	|__|__|	|__|
Other________	|__|	|__|__|	|__|

* Frequency of use (1) weekly (2) monthly (3) annually (4) less than once a year (5) don't know

5.3 Operation and blood transfusion:

5.3.1 Have you ever had a transfusion or surgery? |__| (1) Yes (2) No

If yes, please specify the type of surgical procedure and your age at the time of surgery.

Type of surgery Age

____ |__||__|

____ |__||__|

____ |__||__|

____ |__||__|

5.3.2 Have you had transfusion during the past month? |__| (1) Yes (2) No

5.4 Esophageal reflux

5.4.1have you had a heartburn (post sternum burn) over the past 12 months? |__| 0)No 1)Yes

These symptoms begin at the age of |__| |__| , and the frequency: |__|

(1). Less than once a month (2). probably once a month (3). once a week

(4). 2-3 times a week (5). 4-6 times a week (6). Every day

5.4.2 have you felt sore over the past 12 months? |__| 0)No 1)Yes

These symptoms begin at the age of |__| |__| , and the frequency: |__|

(1). Less than once a month (2). probably once a month (3). once a week

(4). 2-3 times a week (5). 4-6 times a week (6). Every day

5.4.3 The impact of these symptoms on your daily life (including sleep, diet and daily activities):

(1) Sleep |__| (2) Diet |__| (3) Daily activities |__|

Severity classification: (1) Very mild (almost no feeling)

(2) Mild (no feeling if you don't want to)

(3) Moderate (symptoms affect some but not life)

(4) More severe (symptoms affect life)

(5) Severe (symptoms) It has a great impact on this activity and is difficult to carry out

5.4.4 Have you taken antacids in the past 12 months: |__| 0) No 1) Yes, if yes |__|

(1) Ranitidine, Famotidine, Cimetidine (2) Omeprazole, Losec, Pantoprazole, Rabeprazole (3) Others

5.5 Oral hygiene

5.5.1 Have you ever lost any teeth after 20 years old? |__| (1) Yes (2) No

5.5.2 If so, at which age did you loss your first tooth? |__|__| (don’t know: 99)

5.5.3 If so, how many teeth have you lost after 20 years old? |__|__| (don’t know: 99)

5.5.4 Do you have any partial or full denture (plate)? |__| (1) Yes (2) No

5.5.5 If so, how many fixed dentures do you have?

5.5.5.1 Upper: |__|__|

5.5.5.2 Lower: |__|__|

5.5.6 Do you have any removable dentures?

5.5.6.1 Upper: |__| (1) Yes (2) No (3)None

5.5.6.2 Lower: |__| (1) Yes (2) No (3)None

5.5.7 If so, do you usually wear dentures in the day time?

5.5.7.1 Upper: |__| (1) Yes (2) No

5.5.7.2 Lower: |__| (1) Yes (2) No

5.5.8 At which did you start to wear the removable dentures? (None: 98, don't know: 99)

5.5.8.1 Upper: |__|__|

5.5.8.2 Lower: |__|__|

5.5.9 How many teeth have been repaired? (don't count the lost teeth)

5.5.9.1 Upper: |__|__|

5.5.9.2 Lower: |__|__|

5.5.10 How ofter do you brush your teeth every day? |__|

(1) Once per day (2) 2 times per day (3) 3 times per day (4) other ________ (5) never

5.5.11 How often during the last year have you found it uncomfortable to eat particular foods because of problems with your teeth, mouth or dentures? Would you say: |__|

(1) Very often (2) Fairly often (3) Occasionally (4) Hardly ever (5) Never (6) Don't know

5.5.12 If you avoid some food due to a problem in your teeth or gums, which type of food? |__|

(1)Hard and solids foods (2) Cold beverages (3) Both solid foods and cold beverages

(4) Others (5) Don't have any problems

5.6 Family medical history:

Please help me complete the following table listing all your first-degree relatives diagnosed with cancer.

(First-degree relatives are blood relatives including parents, brothers, sisters and children.)

Whether or not: 0 = No 1 = Yes 99= do not know in detail
Cause of death:
1= Heart disease, 2= Cerebrovascular disease, 3= Respiratory infections, 4=HIV, 5=COPD,
6= Diarrheal diseases, 7=TB, 8=Tumor, 9=Accidents, 10= Hypertension, 11=Suicide, 12=other,
99= do not know in detail
Tumor site:
1 = nasopharyngeal carcinoma 2 = other head and neck cancer, 3= esophageal cancer, 4 = stomach cancer, 5 = colorectal cancer, 6 = pancreatic cancer, 7=liver cancer, 8 = lung cancer, 9 = melanoma 10 = breast cancer, 11 =prostate cancer 12 = bladder cancer, 13 = hematological malignancy, 14 = cervical cancer 15=other 99 = do not know in detail
Diagnosis hospital level: 1=Province, 2=City, 3=County, 4=Other, 99= do not know in detail
Diagnosis based on: 1= pathology, 2 = CT, ultrasound , 3 =clinical examination,4 = do not know in detail

5.6.1 Father and mother

Relation	Current age (Survivors)	Age at death (Dead)	Cause of death	Year of death	Ever diagnosed with cancer?	Tumor site	Age at tumor diagnosis	Diagnosis hospital level	Confirmed?	Basis of cancer diagnosis
Father	|__||__|	|__||__|	|__||__|	|__||__||__||__|	|__|	|__||__|	|__||__|	|__||__|	|__|	|__|
Mother	|__||__|	|__||__|	|__||__|	|__||__||__||__|	|__|	|__||__|	|__||__|	|__||__|	|__|	|__|

5.6.2 Brothers

How many brothers do you have?____________（do not know: 99）

Relation	Current age (Survivors)	Age at death (Dead)	Cause of death	Year of death	Ever diagnosed with cancer?	Tumor site	Age at tumor diagnosis	Diagnosis hospital level	Confirmed?	Basis of cancer diagnosis
	|__||__|	|__||__|	|__||__|	|__||__||__||__|	|__|	|__||__|	|__||__|	|__||__|	|__|	|__|
	|__||__|	|__||__|	|__||__|	|__||__||__||__|	|__|	|__||__|	|__||__|	|__||__|	|__|	|__|
	|__||__|	|__||__|	|__||__|	|__||__||__||__|	|__|	|__||__|	|__||__|	|__||__|	|__|	|__|
	|__||__|	|__||__|	|__||__|	|__||__||__||__|	|__|	|__||__|	|__||__|	|__||__|	|__|	|__|
	|__||__|	|__||__|	|__||__|	|__||__||__||__|	|__|	|__||__|	|__||__|	|__||__|	|__|	|__|

5.6.3 Sisters

How many sisters do you have?____________ （do not know: 99）

Relation	Current age (Survivors)	Age at death (Dead)	Cause of death	Year of death	Ever diagnosed with cancer?	Tumor site	Age at tumor diagnosis	Diagnosis hospital level	Confirmed?	Basis of cancer diagnosis
	|__||__|	|__||__|	|__||__|	|__||__||__||__|	|__|	|__||__|	|__||__|	|__||__|	|__|	|__|
	|__||__|	|__||__|	|__||__|	|__||__||__||__|	|__|	|__||__|	|__||__|	|__||__|	|__|	|__|
	|__||__|	|__||__|	|__||__|	|__||__||__||__|	|__|	|__||__|	|__||__|	|__||__|	|__|	|__|
	|__||__|	|__||__|	|__||__|	|__||__||__||__|	|__|	|__||__|	|__||__|	|__||__|	|__|	|__|
	|__||__|	|__||__|	|__||__|	|__||__||__||__|	|__|	|__||__|	|__||__|	|__||__|	|__|	|__|

5.6.4 Sons

How many sons do you have?____________ （do not know: 99）

Relation	Current age (Survivors)	Age at death (Dead)	Cause of death	Year of death	Ever diagnosed with cancer?	Tumor site	Age at tumor diagnosis	Diagnosis hospital level	Confirmed?	Basis of cancer diagnosis
	|__||__|	|__||__|	|__||__|	|__||__||__||__|	|__|	|__||__|	|__||__|	|__||__|	|__|	|__|
	|__||__|	|__||__|	|__||__|	|__||__||__||__|	|__|	|__||__|	|__||__|	|__||__|	|__|	|__|
	|__||__|	|__||__|	|__||__|	|__||__||__||__|	|__|	|__||__|	|__||__|	|__||__|	|__|	|__|
	|__||__|	|__||__|	|__||__|	|__||__||__||__|	|__|	|__||__|	|__||__|	|__||__|	|__|	|__|
	|__||__|	|__||__|	|__||__|	|__||__||__||__|	|__|	|__||__|	|__||__|	|__||__|	|__|	|__|

5.6.5 Daughters

How many daughters do you have ?____________（do not know: 99）

Relation	Current age (Survivors)	Age at death (Dead)	Cause of death	Year of death	Ever diagnosed with cancer?	Tumor site	Age at tumor diagnosis	Diagnosis hospital level	Confirmed?	Basis of cancer diagnosis
	|__||__|	|__||__|	|__||__|	|__||__||__||__|	|__|	|__||__|	|__||__|	|__||__|	|__|	|__|
	|__||__|	|__||__|	|__||__|	|__||__||__||__|	|__|	|__||__|	|__||__|	|__||__|	|__|	|__|
	|__||__|	|__||__|	|__||__|	|__||__||__||__|	|__|	|__||__|	|__||__|	|__||__|	|__|	|__|
	|__||__|	|__||__|	|__||__|	|__||__||__||__|	|__|	|__||__|	|__||__|	|__||__|	|__|	|__|
	|__||__|	|__||__|	|__||__|	|__||__||__||__|	|__|	|__||__|	|__||__|	|__||__|	|__|	|__|

5.6.6 Other relatives (including spouse)

(Including spouse, grandfathers, grandmothers, uncles, aunts, cousins, etc. Report only relatives who were diagnosed with cancer.)

Type of relation	Type of tumor	Age at tumor diagnosis	Diagnosis hospital level	Whether or not tumor was confirmed	Basis of cancer diagnosis
	|__||__|	|__||__|	|__||__|	|__|	|__|
	|__||__|	|__||__|	|__||__|	|__|	|__|
	|__||__|	|__||__|	|__||__|	|__|	|__|
	|__||__|	|__||__|	|__||__|	|__|	|__|

Part VI. History of smoking

6.1 Have you ever smoked tobacco? | __ | (At least one cigarette every 1-3 days for 6 months)
1. Yes, still smoking
2. Formerly smoked, but quit now (at least one year or more)
3. Never smoked (jump to question 6.8)

Please list your status of cigarette, pipe, and cigar smoking at different stages of your life.
(Please recall the times when you changed your smoking habits all your life, and list them separately.)

<table>
<tr><th></th><th>(1) Yes
(2) No</th><th>Age at start</th><th>Age at quiting</th><th>Frequency per day (Cigarettes / cigars: number) (Other tobacco: liang (50 gram)</th><th>Filtered or not? (1 Yes 2 No)</th><th>Deep inhalation or not? (1 Yes 2 No)</th></tr>
<tr><td>6.2 Cigarettes</td><td>|__|</td><td>|__|__|
__	__
__	__
__	__
__	__	</td><td>	__	__
__	__
__	__
__	__
__	__	</td><td>	__	__
__	__
__	__
__	__
__	__	</td><td>	__
__
__
__
__	</td><td>	__
__
__
__
__	</td></tr>
<tr><td>6.3 Self-made cigarettes</td><td>	__	</td><td>	__	__
__	__
__	__	</td><td>	__	__
__	__
__	__	</td><td>	__	__
__	__
__	__	</td><td>	__
__
__	</td><td>	__
__
__	</td></tr>
<tr><td>6.4 Hookah</td><td>	__	</td><td>	__	__
__	__
__	__	</td><td>	__	__
__	__
__	__	</td><td>	__	__
__	__
__	__	</td><td>	__
__
__	</td><td>	__
__
__	</td></tr>
<tr><td>6.5 Pipe</td><td>	__	</td><td>	__	__
__	__
__	__	</td><td>	__	__
__	__
__	__	</td><td>	__	__
__	__
__	__	</td><td>	__
__
__	</td><td>	__
__
__	</td></tr>
<tr><td>6.6 Cigar</td><td>	__	</td><td>	__	__
__	__
__	__	</td><td>	__	__
__	__
__	__	</td><td>	__	__
__	__
__	__	</td><td>	__
__
__	</td><td>	__
__
__	</td></tr>
<tr><td>6.7 Other ___________</td><td>	__	</td><td>	__	__
__	__
__	__	</td><td>	__	__
__	__
__	__	</td><td>	__	__
__	__
__	__	</td><td>	__
__
__	</td><td>	__
__
__	</td></tr>
</table>

*Relation：(1) Parents (2) Spouse (3) Brothers and Sisters (4) Children (5) Other relatives (Including grandparents, uncles, aunts, cousins, etc.) （6）Other<br>† If they smoked cigars or pipes, please use the following formula<br>1 cigarillo = 2 cigarettes<br>1 cigar = 4 cigarettes<br>1 gram of tobacco = 1 cigarette

6.8 When you were a child (before 18 years old), was there someone in your house smoking (anyone of those who lived together)? |__| (1) Yes (2) No

	1	2	3
Relation*			
How many cigarattes did he/she smoke per day? †			
How long have you lived with him/her (before 18 years old)?			

6.9 After you have grown up (after 18 years old), was there someone in your house smoking (anyone of those who lived together)? |__| (1) Yes (2) No

	1	2	3
Relation*			
How many cigarattes did he/she smoke per day? †			
How long have you lived with him/her (after 18 years old)?			

6.10 After you have grown up (after 18 years old), was there someone in your workplace smoking? |__|
(1) Yes (2) No

6.10.1 If yes, how much time have you been exposed to smoking each day ? |__|
(1) less than 30 minutes (2) 30-60 minutes (3) 1-2 hours (4) >2hours

6.10.2 If yes, how many years have you been exposed? |__||__|Years

Part VII. History of alcohol and tea drinking

History of alcohol drinking

7.1 Do you have the habit of drinking alcohol? (At least once a week for 6 months) (1) Yes, (2) No |__|

Type of alcoholic beverage	(1) Yes (2) No	Age at start	Age at quiting	Cups/day or Liang/Day *	Day/week**	Day/month**
7.1.1 Beer	|__|	|__|__| |__|__|	|__|__| |__|__|	|__|__| |__|__|	|__| |__|	|__|__| |__|__|
7.1.2 Yellow rice wine	|__|	|__|__| |__|__|	|__|__| |__|__|	|__|__| |__|__|	|__| |__|	|__|__| |__|__|
7.1.3 Wines	|__|	|__|__| |__|__|	|__|__| |__|__|	|__|__| |__|__|	|__| |__|	|__|__| |__|__|
7.1.4 Fruit wine	|__|	|__|__| |__|__|	|__|__| |__|__|	|__|__| |__|__|	|__| |__|	|__|__| |__|__|
7.1.5 Mild distillate spirits (≤ 38% v/v)	|__|	|__|__| |__|__| |__|__|	|__|__| |__|__| |__|__|	|__|__| |__|__| |__|__|	|__| |__| |__|	|__|__| |__|__| |__|__|
7.1.6 Strong distillate spirits (> 38% v/v)	|__|	|__|__| |__|__| |__|__|	|__|__| |__|__| |__|__|	|__|__| |__|__| |__|__|	|__| |__| |__|	|__|__| |__|__| |__|__|
7.1.7 Imported liquors	|__|	|__|__| |__|__|	|__|__| |__|__|	|__|__| |__|__|	|__| |__|	|__|__| |__|__|

* One cup of Beer, yellow rice wine, wines or fruit wine=200 ml cup

Mild/strong distillate spirits was measured by Liang (1 Liang = 50 ml cup).

** The frequency of drinking alcohol (have to choose one)

History of drinking tea

7.2 Do you have a habit of drinking tea? (At least one cup per day for 6 months) (1) Yes, (2) No |__|

	Size of tea cup<br>(1) small cup<br>(<100 ml)<br>(2) medium cup<br>(100 ml)<br>(3) large cup<br>(>100 ml)	Age at start	Age at quiting	Cups/ per day	Temperature of the tea*	Do you drink strong tea?<br>(1) Yes<br>(2) No
7.2.1 Green tea	|__|	|__|__|	|__|__|	|__|__|	|__|	|__|
7.2.2 Oolong tea	|__|	|__|__|	|__|__|	|__|__|	|__|	|__|
7.2.3 Follow tea	|__|	|__|__|	|__|__|	|__|__|	|__|	|__|
7.2.4 Red tea	|__|	|__|__|	|__|__|	|__|__|	|__|	|__|
7.2.5 Pu'er Tea	|__|	|__|__|	|__|__|	|__|__|	|__|	|__|

* Temperature of the tea (1) Hot - Drink immediately

(2) Warm - Wait for a few minutes before drinking

(3) Mild - Wait for more than 5 minutes before drinking

(4) Cold - Drink after more than 30 minutes or as cold drink

Part VIII Dietary history

8.1. Food and water

8.1.1 What is the source of your drinking water now? |__|

(1) Well (2) Running water (3) Pipe water (4) Other

8.1.2 If pipe water, how many years ago did you start using pipe water? (98 for not using) |__||__|

8.1.3 If pipe water, what was the source of your drinking water before using pipe water?

(1) Well (2) Running water (3) Other_________(4) Have always used pipe water

8.1.4 How do your family preserve food during 2000-2002? |__|

(1) In a refrigerator in a closed box (2) In a refrigerator in an open box (3) Outside a refrigerator in a closed box (4) Outside a refrigerator in an open box (5) In a plastic bag (6) In a cotton napery

8.1.5 How do your family prepare your food during 2000-2002?

(0) Never (1) sometimes (2) Often (3) Most of the times

	Fresh vegetables	Meat	Fresh fish	Salted fish
Fried	|__|	|__|	|__|	|__|
Deep fried	|__|	|__|	|__|	|__|
Braised in brown sauce	|__|	|__|	|__|	|__|
Boiled	|__|	|__|	|__|	|__|
Stir-fried	|__|	|__|	|__|	|__|
Steamed	|__|	|__|	|__|	|__|
Stewed	|__|	|__|	|__|	|__|
Other	|__|	|__|	|__|	|__|

8.2 Preserved vegetables

8.2.1 Have you ever eaten pickled vegetables (such as Chinese sauerkraut, pickles, Vinegar pickled, etc.)?

|__| 1)Yes 2)No.

If yes, please answer the following questions:

8.2.2 Do you make your own preserved vegetables or buy them in the store?

|__| 1) Make it yourself 2) Buy it in the store.

8.2.3 How many times do you eat preseved vegetables every day (week, month, year)?

At the end of the 60 s: _____times/day; _____times/week; _____times/month; _____times/year;

At the end of the 70 s: _____times/day； _____times/week； _____times/month； _____times/year；

At the end of the 80 s: _____times/day； _____times/week； _____times/month； _____times/year；

At the end of the 90 s: _____times/day； _____times/week； _____times/month； _____times/year；

8.2.4 What vegetables do you usually use to make preseved vegetables?________

1. Garlic 2. Yellow turnip 3. Green turnip 4. Chinese cabbage 5. Cucumber 6. Green vegetables 7. Fragrant-flowered garlic 8. Green Chinese onion 9.Tomato 10. hot pepper 11.eggplant.

8.2.5 How long have the pickles you eat been preserved?|__||__||__| days；

How long will it take to eat it all? |__||__||__|days.

8.2.6.1 In the past, how long did you eat preseved vegetables a year on average? |__||__|months/year；

In what season: |__||__||__||__|

1) Summer 2) Autumn 3) Winter 4) Spring.

8.2.6.2 How long do you eat preseved vegetables a year on average now? ||__||__|months/year；

In what season: |__||__||__||__|

1) Summer 2) Autumn 3) Winter 4) Spring.

8.2.7 Is the preseved vegetables you eat mildewed?

Please answer: |__| 1) Often 2) Sometimes 3) Occasionally 4) Never 5) Don't know.

8.3 The following information is about the eating habits of 10 years ago (2000-2002)

- If you do not eat or rarely eat (<1 time per year), draw a tick in the never column.
- If the number of times of eating is greater than 1 time per year, the number of times should be written in the corresponding column according to the frequency of consumption and fill in the corresponding average consumption per time. For example, someone who spends 2 meals per day between 2000 and 2002. In the " times per day" column filled with 2.
- Note: the average unit of consumption for each consumption, such as jin, liang, one number, piece, cup, spoon (see photo) and so on. In the above example, if you eat three liang each time, in the "average per consumption" column filled with three liang.

	**Food name**	**2000-2002**					
		Never	Times per year	Times per month	Times per week	Times per day	Average consumption per time
**Staple food**	Rice						liang
	Steamed bread with white flour (steamed roll)						liang
	Vegetable porridge, acidic-Gruel						liang
	Doughball						liang
	Sesame seed cake(Steamed pancake, deep-fried dough cake)						liang
	Noodles						liang
	Steamed stuffed bun						liang
	Sweet potato						liang
	Wonton						liang
	Porridge (highland barley, corn)						liang
	Oats, buckwheat						liang
	Bread, toast						pcs
	Deep-fried dough sticks, spring roll						pcs
	Rice ball with vegetable inside						liang

	Food name	2000-2002					
		Never	Times per year	Times per month	Times per week	Times per day	Average consumption per time
**Pickled food**	Salted fish						liang
	Preserved vegetables (including dried radishes, soy bean, salted cagetables, Chinese sauerkraut, meicai, mustard, etc)						liang
	Fermented bean curd						pcs
	Preserved egg						pcs
	Salted egg						pcs
	Stinky tofu						liang
	Dried pork floss						liang
	Dried pork slice						liang
	Sausage, ham, preserved duck, etc						liang
	Others___________						
**Eggs**	Egg						pcs
**Fresh meat**	Pork, beef, mutton, dog meat, rabbit meat						liang
	Chicken, duck, goose, pigeon						liang
	Pluck						liang
	Others___________						liang
**Fresh aquatic products**	Fresh freshwater fish						liang
	Fresh sea fish						liang

	Food name	2000-2002					
		Never	Times per year	Times per month	Times per week	Times per day	Average consumption per time
	Shrimp, crab						liang
	Shellfish, snails						liang
	Others___________						
**Milk and dairy products**	Fresh milk and boxed milk						Middle cup
	Milk powder						Medium sized spoonful
	Yogurt						Middle cup
	Others___________						
**Dessert and nuts**	Western-style dessert(Cream bun, cake, egg tart, etc)						pcs
	All kinds of biscuits						pcs
	Nuts (peanuts, cashews, almonds, walnuts, etc)						liang
**Beans and soy products**	Dried beans (soybeans, mung beans, red beans, etc)						liang
	Soybean milk, bean curd jelly						Middle cup
	Tofu						pcs
	Dried bean curd\Chiba tofu\bean curd strips						liang
	Bean sprout						liang
	Others___________						
Fresh vegetables and fruits are mostly seasonal. We are now asking about your consumption of vegetables or fruits when they are on the market. (Including frequency and average consumption every time)							
**Fresh**	Green leafy vegetables						liang

	Food name	2000-2002					
		Never	Times per year	Times per month	Times per week	Times per day	Average consumption per time
**vegetables, fungi and algae**	Pumpkin						liang
	Melons (cucumber, white gourd, tower gourd, bitter gourd, etc)						liang
	White radish						liang
	Radish						liang
	Chinese cabbage						liang
	Edamame						liang
	Bean (cowpea, lentil)						liang
	Eggplant						liang
	Tomato						liang
	Green, red pepper, bell pepper						liang
	Corn						pcs
	Cabbage						liang
	Celery						liang
	Lettuce, asparagus lettuce						
	Lotus root, taro						liang
	Garlic sprout						liang
	Onions						liang
	Spring onions, scallions, garlic						liang

	Food name	2000-2002					
		Never	Times per year	Times per month	Times per week	Times per day	Average consumption per time
	All kinds of fresh mushrooms, agaric						liang
	Kelp						liang
	Nori						liang
	Others______						
**Fresh fruit**	Apple						pcs
	Banana						pcs
	Orange						pcs
	Mandarin orange						pcs
	Peach						pcs
	Pineapple						pcs
	Ginkgo						liang
	Pears						pcs
	Cantaloup						pcs
	Grape						pcs
	Persimmon, dried persimmon						pcs
	Watermelon						liang
	Others__________						liang

8.4 Cooking oil

8.4.1 About 10 years ago (2000-2002), how many_____ people in your family, and how much cooking oil _____ kg/month was consumed by the whole family（Don’t know 99）

8.4.2 Types of cooking oil？ |__||__| (at most 2 types)

(1) peanut oil (2) rapeseed oil (3) blending oil (4) Soybean oil (5) Other______ (6)Don’t know

Part IX：Reproductive history (Applies only for women)

8.1 Have you ever been pregnant? (1) Yes，_____ times (2) No

8.2 Age at first pregnancy ____ years old

8.3 The outcome of first pregnancy (1) natural delivery (2) stillbirth (3) abortion (4) other ____

8.4 Total number of deliveries ________(including still births)

8.5 Have you had menopause? (1) Yes, menopausal at : ______years old (2) No

Part X. Interviewer's evaluation

Interview location: |__| (1) Interviewee's home (2) hospital (3) others___________________

Interview end time 201_ year __ month __ day ___hour ___minute

Language used for interview |__| (1) Taixing dialect (2) Mandarin (3) Other_______________

Evaluation of the interview: (1) Reliable (2) Unreliable

Part I |__|, if answer is 'unreliable', the reason |__| _______________________________________

Part II |__|, if answer is 'unreliable', the reason |__| ______________________________________

Part III |__|, if answer is 'unreliable', the reason |__| ____________________________________

Part IV |__|, if answer is 'unreliable', the reason |__| _____________________________________

Part V |__|, if answer is 'unreliable', the reason |__| ______________________________________

Part VI |__|, if answer is 'unreliable', the reason |__| _____________________________________

Part VII |__|, if answer is 'unreliable', the reason |__| ____________________________________

Part VIII |__|, if answer is 'unreliable', the reason |__| ___________________________________

Part IX |__|, if answer is 'unreliable', the reason |__| _____________________________________

Reasons

(1) The subject did not want to collaborate, and did not want to answer questions

(2) The subject could not recall clearly, and provided conflicting answers

(3) Other

Name of interviewer： _____________________________________

Signature of interviewer： __________________________________
