# Supplementary material for: A nomogram for screening esophageal squamous cell carcinoma based on environmental risk factors in a high-incidence area of China: a population-based case-control study
Source: BMC Cancer. 2021 Mar 31;21:343. doi: 10.1186/s12885-021-08053-7 (PMC8011400; doi:10.1186/s12885-021-08053-7)
Supplement: Supplementary file 2 — Additional file 2: Table S1. The age distribution of local residents in Taixing city and the weight of controls in our data analysis. [file 12885_2021_8053_MOESM2_ESM.docx]

**Table S1.** The age distribution of local residents in Taixing city and the weight of controls in our data analysis.

| **Age group**  **(Year)** | **The sixth census of Taixing City (2010)** | | | **The controls**  **in our study ^a^** | | **The weight in data analysis ^c^** | |
| --- | --- | --- | --- | --- | --- | --- | --- |
|  | **Total**  **(N=573 706)**  **N (%)** | **Men**  **(N=266 209)**  **N (%)** | **Women**  **(N=307 497)**  **N (%)** | **Men**  **(N=1338)**  **N (%)** | **Women**  **(N=610)**  **N (%)** | **Men** | **Women** |
| 40-44 | 111 180 (19.38) | 40 645 (15.27) | 70 535 (22.94) | 11 (0.82) | 9 (1.48) | 18.57 | 15.55 |
| 45-49 | 87 719 (15.29) | 40 645 (15.27) | 47 074 (14.85) | 41 (3.06) | 20 (3.28) | 4.98 | 4.67 |
| 50-54 | 74 096 (12.92) | 36 454 (13.69) | 37 642 (11.87) | 71 (5.31) | 33 (5.41) | 2.58 | 2.26 |
| 55-59 | 83 164 (14.5) | 42 151 (15.83) | 41 013 (12.94) | 204 (15.25) | 57 (9.34) | 1.04 | 1.43 |
| 60-64 | 72 595 (12.65) | 37 899 (14.24) | 34 696 (10.94) | 270 (20.18) | 83 (13.61) | 0.71 | 0.83 |
| 65-69 | 51 910 (9.05) | 26 948 (10.12) | 24 962 (7.87) | 304 (22.72) | 123 (20.16) | 0.45 | 0.40 |
| 70-74 | 41 411 (7.22) | 20 054 (7.53) | 21 357 (6.74) | 238 (17.79) | 133 (21.8) | 0.42 | 0.32 |
| 75-79 | 30 685 (5.35) | 13 378 (5.03) | 17 307 (5.46) | 152 (11.36) | 99 (16.23) | 0.44 | 0.35 |
| 80-84^b^ | 20 946 (3.65) | 8 035 (3.02) | 12 911 (4.07) | 47 (3.51) | 53 (8.69) | 0.86 | 0.48 |
| ^a^ The observations with missing information were excluded.  ^b^ The controls with 85 years old in our study were changed to the age group with 80-84.  ^c^ The weight was calculated by dividing the age proportion of local census by the age proportion of the controls in our study. | | | | | | | |
