# Supplementary material for: A nomogram for screening esophageal squamous cell carcinoma based on environmental risk factors in a high-incidence area of China: a population-based case-control study
Source: BMC Cancer. 2021 Mar 31;21:343. doi: 10.1186/s12885-021-08053-7 (PMC8011400; doi:10.1186/s12885-021-08053-7)
Supplement: Supplementary file 3 — Additional file 3: Table S2. OR with 95%CIs and nomogram points of candidate ESCC risk factors. [file 12885_2021_8053_MOESM3_ESM.docx]

**Table S2.** OR with 95%CIs and nomogram points of candidate ESCC risk factors. **^a^**

| **Variables** | **Men** | |  | | **Women** | |
| --- | --- | --- | --- | --- | --- | --- |
|  | **OR (95% CI)** | **Points ^b^** | | **OR (95% CI)** | | **Points ^b^** |
| **Education** |  |  | |  | |  |
| Illiteracy | 1.83(1.23~2.71) | 3.9 | | 3.57 (0.71~17.86) | | 7.0 |
| Primary school | 1.25(0.90~1.75) | 1.5 | | 2.46 (0.49~12.36) | | 4.9 |
| Junior high school | 1.00(0.72~1.40) | 0 | | 1.08 (0.20~5.92) | | 0.4 |
| High school and above | 1.00 (reference) | 0 | | 1.00 (reference) | | 0 |
| **Family wealth score** |  |  | |  | |  |
| Q1-lowest | 2.82(2.04~3.90) | 6.7 | | 2.41 (1.45~4.01) | | 4.8 |
| Q2 | 1.96(1.39~2.75) | 4.3 | | 1.71 (1.02~2.88) | | 2.9 |
| Q3 | 1.81(1.31~2.49) | 3.8 | | 1.86 (1.12~3.08) | | 3.4 |
| Q4 | 1.66(1.20~2.30) | 3.3 | | 1.45 (0.87~2.43) | | 2.0 |
| Q5 | 1.00 (reference) | 0 | | 1.00 (reference) | | 0 |
| **BMI at 10 years ago ^c^** |  |  | |  | |  |
| <18.5 (Underweight) | 1.76(0.99~3.12) | 3.6 | | 2.26 (1.03~4.96) | | 4.4 |
| [18.5, 24) (Normal) | 1.25(0.81~1.92) | 1.4 | | 1.66 (0.83~3.31) | | 2.8 |
| [24, 28) (Overweight) | 1.00(0.63~1.58) | 0 | | 1.81 (0.89~3.69) | | 3.2 |
| ≥ 28 (Obese) | 1.00 (reference) | 0 | | 1.00 (reference) | | 0 |
| **Adult height (cm, male \| female)** |  |  | |  | |  |
| ≤ 162 \| ≤ 152 | 1.00 (reference) | 0 | | 1.00 (reference) | | 0 |
| (162, 170] \| (152, 156] | 2.86(2.26~3.62) | 6.8 | | 4.37 (2.97~6.42) | | 8.0 |
| (170, 174] \| (156, 160] | 3.91(2.77~5.52) | 8.8 | | 5.12 (3.45~7.59) | | 8.9 |
| >174 \| >160 | 4.74(3.39~6.63) | 10.0 | | 6.24 (3.91~9.95) | | 10.0 |
| **Frequency of tooth brushing per day** |  |  | |  | |  |
| < 2 | 1.00 (reference) | 0 | | 1.00 (reference) | | 0 |
| ≥ 2 | 1.98(1.59~2.46) | 4.4 | | 2.35 (1.71~3.23) | | 4.7 |
| **Sum of missing and filled teeth** |  |  | |  | |  |
| None | ─ | ─ | | 1.00 (reference) | | 0 |
| < 6 |  |  | | 1.20 (0.79~1.83) | | 1.0 |
| ≥ 6 |  |  | | 1.77 (1.19~2.63) | | 3.1 |
| **Smoking pack-years** |  |  | |  | |  |
| Never | 1.00 (reference) | 0.3 | | ─ | | ─ |
| ≤ 30 | 0.95(0.73~1.24) | 0 | |  | |  |
| > 30 | 1.14(0.87~1.49) | 1.2 | |  | |  |
| **Alcohol drinking intensity (g/day)** |  |  | |  | |  |
| Never | 1.00 (reference) | 0 | | ─ | | ─ |
| ≤ 80 | 2.06(1.62~2.62) | 4.6 | |  | |  |
| > 80 | 2.35(1.86~2.97) | 5.5 | |  | |  |
| **Tea drinking temperature ^d^** |  |  | |  | |  |
| Never | 1.00 (reference) | 0 | | ─ | | ─ |
| Warm | 1.20(0.91~1.57) | 1.2 | |  | |  |
| Hot | 1.40(1.08~1.82) | 2.2 | |  | |  |
| Very Hot | 2.03(1.43~2.89) | 4.6 | |  | |  |
| **Family history of esophageal cancer among first-degree relatives** |  |  | |  | |  |
| No | 1.00 (reference) | 0 | | 1.00 (reference) | | 0 |
| Yes | 2.27(1.83~2.81) | 5.3 | | 2.11 (1.53~2.91) | | 4.1 |
| ^a^ Adjusted for education, family wealth score, BMI, sum of missing and filled teeth, tooth brushing times, family history of esophageal cancer, and further adding smoking pack-years, alcohol consumption intensity, tea drinking temperature in men (all variables are categorized as shown in Table 1).  ^b^ The assignment points of each variable based on nomogram predictive model. | | | | | | |
