# Supplementary material for: A nomogram for screening esophageal squamous cell carcinoma based on environmental risk factors in a high-incidence area of China: a population-based case-control study
Source: BMC Cancer. 2021 Mar 31;21:343. doi: 10.1186/s12885-021-08053-7 (PMC8011400; doi:10.1186/s12885-021-08053-7)
Supplement: Supplementary file 4 — Additional file 4: Table S3. OR with 95%CIs and nomogram points of candidate ESCC risk factors by a weighted analysis. [file 12885_2021_8053_MOESM4_ESM.docx]

**Table S3.** OR with 95%CIs and nomogram points of candidate ESCC risk factors by a weighted analysis **^a^**.

| **Variables** | **Men** | |  | | **Women** | |
| --- | --- | --- | --- | --- | --- | --- |
|  | **OR (95% CI)** | **Points ^b^** | | **OR (95% CI)** | | **Points ^b^** |
| **Age group** |  |  | |  | |  |
| 40-49 | 0.11(0.06~0.20) | 0 | | 0.03(0.01~0.09) | | 0 |
| 50-59 | 0.48(0.37~0.63) | 6.4 | | 0.20(0.12~0.32) | | 5.1 |
| 60-69 | 1.00 (reference) | 9.6 | | 1.00 (reference) | | 9.2 |
| 70-79 | 1.11(0.86~1.42) | 10.0 | | 1.34(0.94~1.91) | | 10.0 |
| 80-84 | 0.69(0.41~1.16) | 8.0 | | 0.69(0.40~1.18) | | 8.3 |
| **Education** |  |  | |  | |  |
| Illiteracy | 2.16 (1.42~3.28) | 3.4 | | 3.19 (0.68~15.08) | | 3.3 |
| Primary school | 1.37 (0.96~1.96) | 1.4 | | 1.92 (0.4~9.16) | | 2.0 |
| Junior high school | 1.15 (0.8~1.64) | 0.6 | | 0.88 (0.17~4.57) | | 0 |
| High school and above | 1.00 (reference) | 0 | | 1.00 (reference) | | 0.3 |
| **Family wealth score** |  |  | |  | |  |
| Q1-lowest | 2.93 (2.07~4.16) | 4.7 | | 2.79 (1.61~4.83) | | 2.6 |
| Q2 | 1.83 (1.27~2.64) | 2.6 | | 1.84 (1.05~3.22) | | 1.6 |
| Q3 | 1.82 (1.3~2.57) | 2.6 | | 1.91 (1.12~3.26) | | 1.7 |
| Q4 | 1.69 (1.19~2.38) | 2.3 | | 1.63 (0.94~2.8) | | 1.3 |
| Q5 | 1.00 (reference) | 0 | | 1.00 (reference) | | 0 |
| **Adult height (cm, male \| female)** |  |  | |  | |  |
| ≤ 162 \| ≤ 152 | 1.00 (reference) | 0 | | 1.00 (reference) | | 0 |
| (162, 170] \| (152, 156] | 2.72 (2.12~3.49) | 4.4 | | 4.31 (2.87~6.48) | | 3.8 |
| (170, 174] \| (156, 160] | 4.15 (2.83~6.09) | 6.2 | | 5.04 (3.32~7.66) | | 4.2 |
| >174 \| >160 | 4.32 (2.98~6.26) | 6.4 | | 5.88 (3.57~9.7) | | 4.6 |
| **Frequency of tooth brushing per day** |  |  | |  | |  |
| < 2 | 1.00 (reference) | 0 | | 1.00 (reference) | | 0 |
| ≥ 2 | 2.02 (1.59~2.56) | 3.1 | | 2.29 (1.64~3.2) | | 2.1 |
| **Sum of missing and filled teeth** |  |  | |  | |  |
| None | 1.00 (reference) | 0 | | 1.00 (reference) | | 0 |
| < 6 | 1.01 (0.78~1.30) | 0.1 | | 1.26 (0.79~1.99) | | 0.6 |
| ≥ 6 | 1.47 (1.11~1.96) | 1.6 | | 1.73 (1.1~2.72) | | 1.4 |
| **Smoking pack-years** |  |  | |  | |  |
| Never | 1.00 (reference) | 0.1 | | ─ | | ─ |
| ≤ 30 | 0.98 (0.74~1.31) | 0 | |  | |  |
| > 30 | 1.23 (0.93~1.64) | 1.0 | |  | |  |
| **Alcohol drinking intensity (g/day)** |  |  | |  | |  |
| Never | 1.00 (reference) | 0 | | ─ | | ─ |
| ≤ 80 | 2.13 (1.64~2.75) | 3.3 | |  | |  |
| > 80 | 2.46 (1.90~3.17) | 3.9 | |  | |  |
| **Tea drinking temperature** |  |  | |  | |  |
| Never | 1.00 (reference) | 0 | | ─ | | ─ |
| Warm | 1.24 (0.92~1.66) | 0.9 | |  | |  |
| Hot | 1.36 (1.01~1.84) | 1.3 | |  | |  |
| Very Hot | 2.18 (1.48~3.21) | 3.9 | |  | |  |
| **Family history of esophageal cancer among first-degree relatives** |  |  | |  | |  |
| No | 1.00 (reference) | 0 | | 1.00 (reference) | | 0 |
| Yes | 2.11 (1.66~2.68) | 3.3 | | 2.25 (1.6~3.16) | | 2.1 |
| OR, odds ratio; CI, confidence interval.  ^a^ Adjusted for age group, education, family wealth score, adult height, sum of missing filled teeth, tooth brushing times, and family history of esophageal cancer, and further adding smoking pack-years, alcohol consumption intensity, tea drinking temperature in men (all variables are categorized as shown in this table).  ^b^ The assignment points of each variable based on nomogram predictive model. | | | | | | |
